# 4-Methyl-3-nitro­benzonitrile

**DOI:** 10.1107/S1600536808027414

**Published:** 2008-09-06

**Authors:** Li-Jing Cui, Jing Dai

**Affiliations:** a Ordered Matter Science Research Center, College of Chemistry and Chemical, Engineering, Southeast University, Nanjing 210096, People’s Republic of China.

## Abstract

In the title compound, C_8_H_6_N_2_O_2_, the nitro group is rotated by 23.2 (3)° out of the plane of the benzene ring. The crystal structure is stabilized by van der Waals inter­actions.

## Related literature

For the chemistry of nitrile derivatives, see: Xiong *et al.* (2002 ▶); Jin *et al.* (1994 ▶); Brewis *et al.* (2003 ▶); Dunica *et al.* (1991 ▶). For related literature, see: Fu & Zhao (2007 ▶); Liang & Wang, (2008 ▶).
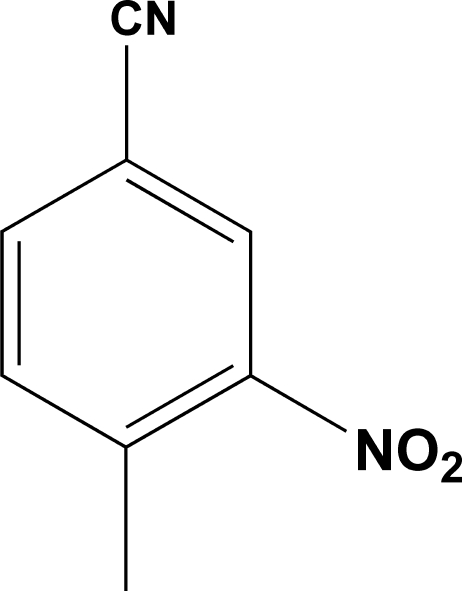


## Experimental

### 

#### Crystal data

C_8_H_6_N_2_O_2_
*M* *_r_* = 162.15
Monoclinic, 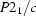

*a* = 3.9088 (8) Å
*b* = 13.576 (3) Å
*c* = 14.819 (4) Å
β = 99.13 (3)°
*V* = 776.4 (3) Å^3^
*Z* = 4
Mo *K*α radiation
μ = 0.10 mm^−1^
*T* = 298 (2) K
0.35 × 0.30 × 0.1 mm

#### Data collection

Rigaku Mercury2 diffractometer
Absorption correction: multi-scan (*CrystalClear*; Rigaku, 2005 ▶) *T*_min_ = 0.965, *T*_max_ = 0.990
7589 measured reflections
1761 independent reflections
1336 reflections with *I* > 2σ(*I*)
*R*_int_ = 0.037

#### Refinement

*R*[*F* ^2^ > 2σ(*F* ^2^)] = 0.068
*wR*(*F* ^2^) = 0.208
*S* = 1.10
1753 reflections
109 parameters
H-atom parameters constrained
Δρ_max_ = 0.33 e Å^−3^
Δρ_min_ = −0.28 e Å^−3^

### 

Data collection: *CrystalClear* (Rigaku, 2005 ▶); cell refinement: *CrystalClear*; data reduction: *CrystalClear*; program(s) used to solve structure: *SHELXS97* (Sheldrick, 2008 ▶); program(s) used to refine structure: *SHELXL97* (Sheldrick, 2008 ▶); molecular graphics: *SHELXTL* (Sheldrick, 2008 ▶); software used to prepare material for publication: *SHELXTL*.

## Supplementary Material

Crystal structure: contains datablocks I, global. DOI: 10.1107/S1600536808027414/wk2090sup1.cif

Structure factors: contains datablocks I. DOI: 10.1107/S1600536808027414/wk2090Isup2.hkl

Additional supplementary materials:  crystallographic information; 3D view; checkCIF report

## Figures and Tables

**Fig. 1.**
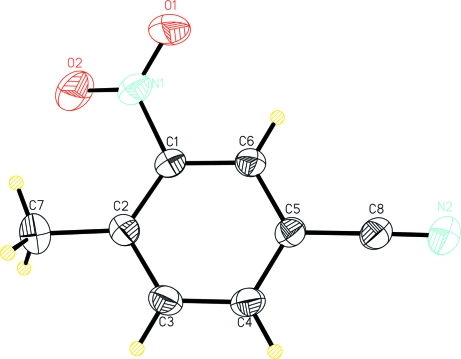
A view of the molecular structure of the title compound, with the atomic numbering scheme. Displacement ellipsoids are drawn at the 30% probability level. H atoms are represented by spheres of arbitrary radius.

## supplementary crystallographic information

### Comment

Nitrile derivatives have found a wide range of applications in industry and coordination chemistry as ligands. For example, phthalonitriles have been used as starting materials for phthalocyanines (Jin *et al.*, 1994), which are important components for dyes, pigments, gas sensors, optical limiters and liquid crystals, and which are also used in medicine, as singlet oxygen photosensitisers for photodynamic therapy (Brewis *et al.*, 2003). Also, nitrile compounds are the precursors of tetrazole complexes (Dunica *et al.*(1991); Xiong *et al.*(2002)). Recently, a series of benzonitrile compounds have been reported (Fu & Zhao, 2007; Liang & Wang, 2008). As an extension of these studies on structural characterization, we report here the crystal structure of the title compound, *p*-methyl-*m*-nitrobenzonitrile.

The crystal data show that in the title compound (Fig. 1), the benzene ring and the nitro group are not coplanar, they are twisted with respect to each other by torsion angles of O1—N1—C1—C6 (-23.2 (4)°) and O2—N1—C1—C2 (-25.6 (3)°); the nitrile group C8≡N2 bond length of 1.144 (3) Å is within the normal range. The crystal structure is stabilized only by van der Waals interactions.

### Experimental

The purchased *p*-methyl-*m*-nitrobenzonitrile (3 mmol, 486.44 mg) was dissolved in chloroform (20 ml) and evaporated in air, affording colorless block crystals of this compound suitable for X-ray analysis.

### Refinement

All H atoms bonded to C atoms were fixed geometrically and treated as riding with C—H = 0.93 Å (aromatic), C—H = 0.96 Å (methyl), with *U*_iso_(H) = 1.2*U*_eq_(aromatic C) and *U*_iso_(H) = 1.5*U*_eq_(methyl C).

### Crystal data

**Table:**

C_8_H_6_N_2_O_2_	*F*(000) = 336
*M**_r_* = 162.15	*D*_x_ = 1.387 Mg m^−^^3^
Monoclinic, *P*2_1_/*c*	Mo *K*α radiation, λ = 0.71073 Å
Hall symbol: -P 2ybc	Cell parameters from 1764 reflections
*a* = 3.9088 (8) Å	θ = 3.1–27.6°
*b* = 13.576 (3) Å	µ = 0.10 mm^−^^1^
*c* = 14.819 (4) Å	*T* = 298 K
β = 99.13 (3)°	Block, colourless
*V* = 776.4 (3) Å^3^	0.35 × 0.30 × 0.1 mm
*Z* = 4	

### Data collection

**Table:**

Rigaku Mercury2 diffractometer	1761 independent reflections
Radiation source: fine-focus sealed tube	1336 reflections with *I* > 2σ(*I*)
graphite	*R*_int_ = 0.037
Detector resolution: 13.6612 pixels mm^-1^	θ_max_ = 27.5°, θ_min_ = 3.0°
ω scans	*h* = −5→5
Absorption correction: multi-scan (CrystalClear; Rigaku/MSC, 2005)	*k* = −17→17
*T*_min_ = 0.965, *T*_max_ = 0.990	*l* = −19→19
7589 measured reflections	

### Refinement

**Table:**

Refinement on *F*^2^	Primary atom site location: structure-invariant direct methods
Least-squares matrix: full	Secondary atom site location: difference Fourier map
*R*[*F*^2^ > 2σ(*F*^2^)] = 0.068	Hydrogen site location: inferred from neighbouring sites
*wR*(*F*^2^) = 0.208	H-atom parameters constrained
*S* = 1.10	*w* = 1/[σ^2^(*F*_o_^2^) + (0.1036*P*)^2^ + 0.2336*P*] where *P* = (*F*_o_^2^ + 2*F*_c_^2^)/3
1753 reflections	(Δ/σ)_max_ < 0.001
109 parameters	Δρ_max_ = 0.33 e Å^−^^3^
0 restraints	Δρ_min_ = −0.28 e Å^−^^3^

### Special details

**Table:**

Geometry. All e.s.d.'s (except the e.s.d. in the dihedral angle between two l.s. planes) are estimated using the full covariance matrix. The cell e.s.d.'s are taken into account individually in the estimation of e.s.d.'s in distances, angles and torsion angles; correlations between e.s.d.'s in cell parameters are only used when they are defined by crystal symmetry. An approximate (isotropic) treatment of cell e.s.d.'s is used for estimating e.s.d.'s involving l.s. planes.
Refinement. Refinement of *F*^2^ against ALL reflections. The weighted *R*-factor *wR* and goodness of fit *S* are based on *F*^2^, conventional *R*-factors *R* are based on *F*, with *F* set to zero for negative *F*^2^. The threshold expression of *F*^2^ > σ(*F*^2^) is used only for calculating *R*-factors(gt) *etc*. and is not relevant to the choice of reflections for refinement. *R*-factors based on *F*^2^ are statistically about twice as large as those based on *F*, and *R*- factors based on ALL data will be even larger.

### Fractional atomic coordinates and isotropic or equivalent isotropic displacement parameters (Å^2^)

**Table:**

	*x*	*y*	*z*	*U*_iso_*/*U*_eq_	
O1	1.0921 (7)	0.79875 (18)	0.59741 (14)	0.1001 (9)	
O2	1.3658 (7)	0.89476 (18)	0.52048 (17)	0.0932 (8)	
N1	1.1627 (5)	0.82762 (16)	0.52533 (14)	0.0584 (6)	
N2	0.5214 (8)	0.45178 (18)	0.38967 (18)	0.0803 (8)	
C1	1.0021 (5)	0.77768 (15)	0.44045 (14)	0.0444 (5)	
C8	0.6195 (7)	0.53049 (18)	0.38358 (16)	0.0568 (6)	
C2	0.9631 (5)	0.82620 (16)	0.35611 (15)	0.0462 (5)	
C6	0.8914 (6)	0.68242 (16)	0.45126 (14)	0.0470 (5)	
H6	0.9185	0.6535	0.5088	0.056*	
C4	0.6963 (6)	0.67560 (17)	0.28898 (15)	0.0530 (6)	
H4	0.5942	0.6412	0.2374	0.064*	
C5	0.7388 (6)	0.63055 (16)	0.37444 (15)	0.0463 (5)	
C3	0.8057 (7)	0.77130 (18)	0.28090 (16)	0.0559 (6)	
H3	0.7738	0.8004	0.2234	0.067*	
C7	1.0660 (8)	0.93151 (18)	0.3406 (2)	0.0672 (7)	
H7A	1.1700	0.9601	0.3976	0.101*	
H7B	0.8641	0.9687	0.3157	0.101*	
H7C	1.2293	0.9326	0.2985	0.101*	

### Atomic displacement parameters (Å^2^)

**Table:**

	*U*^11^	*U*^22^	*U*^33^	*U*^12^	*U*^13^	*U*^23^
O1	0.145 (2)	0.1075 (18)	0.0430 (11)	−0.0405 (15)	0.0006 (12)	−0.0087 (11)
O2	0.0966 (17)	0.0881 (15)	0.0906 (16)	−0.0397 (13)	0.0017 (12)	−0.0237 (12)
N1	0.0605 (12)	0.0593 (12)	0.0523 (12)	−0.0039 (10)	−0.0011 (9)	−0.0117 (9)
N2	0.106 (2)	0.0562 (14)	0.0755 (16)	−0.0203 (13)	0.0039 (14)	−0.0061 (11)
C1	0.0410 (10)	0.0476 (12)	0.0436 (11)	0.0013 (9)	0.0041 (8)	−0.0064 (9)
C8	0.0662 (15)	0.0501 (13)	0.0521 (14)	−0.0036 (11)	0.0029 (11)	−0.0063 (10)
C2	0.0447 (11)	0.0456 (11)	0.0496 (12)	0.0061 (9)	0.0119 (9)	0.0026 (9)
C6	0.0517 (12)	0.0478 (12)	0.0402 (11)	0.0010 (9)	0.0034 (9)	0.0011 (9)
C4	0.0619 (14)	0.0521 (13)	0.0417 (12)	0.0062 (10)	−0.0014 (10)	−0.0048 (9)
C5	0.0483 (12)	0.0446 (11)	0.0452 (12)	0.0015 (9)	0.0049 (8)	−0.0036 (9)
C3	0.0708 (16)	0.0543 (13)	0.0415 (12)	0.0083 (11)	0.0058 (10)	0.0059 (9)
C7	0.0707 (17)	0.0504 (14)	0.0807 (19)	−0.0005 (12)	0.0125 (14)	0.0096 (12)

### Geometric parameters (Å, °)

**Table:**

O1—N1	1.210 (3)	C6—C5	1.390 (3)
O2—N1	1.218 (3)	C6—H6	0.9300
N1—C1	1.478 (3)	C4—C3	1.379 (3)
N2—C8	1.144 (3)	C4—C5	1.392 (3)
C1—C6	1.381 (3)	C4—H4	0.9300
C1—C2	1.400 (3)	C3—H3	0.9300
C8—C5	1.450 (3)	C7—H7A	0.9600
C2—C3	1.400 (3)	C7—H7B	0.9600
C2—C7	1.513 (3)	C7—H7C	0.9600
			
O1—N1—O2	122.4 (2)	C3—C4—H4	120.0
O1—N1—C1	118.5 (2)	C5—C4—H4	120.0
O2—N1—C1	119.1 (2)	C6—C5—C4	119.7 (2)
C6—C1—C2	123.55 (19)	C6—C5—C8	120.1 (2)
C6—C1—N1	115.43 (19)	C4—C5—C8	120.2 (2)
C2—C1—N1	121.0 (2)	C4—C3—C2	122.4 (2)
N2—C8—C5	178.9 (3)	C4—C3—H3	118.8
C3—C2—C1	115.6 (2)	C2—C3—H3	118.8
C3—C2—C7	118.4 (2)	C2—C7—H7A	109.5
C1—C2—C7	125.9 (2)	C2—C7—H7B	109.5
C1—C6—C5	118.75 (19)	H7A—C7—H7B	109.5
C1—C6—H6	120.6	C2—C7—H7C	109.5
C5—C6—H6	120.6	H7A—C7—H7C	109.5
C3—C4—C5	119.9 (2)	H7B—C7—H7C	109.5
			
O1—N1—C1—C6	−23.2 (3)	N1—C1—C6—C5	−179.76 (19)
O2—N1—C1—C6	155.4 (2)	C1—C6—C5—C4	−0.9 (3)
O1—N1—C1—C2	155.8 (2)	C1—C6—C5—C8	−179.9 (2)
O2—N1—C1—C2	−25.6 (3)	C3—C4—C5—C6	0.0 (3)
C6—C1—C2—C3	−0.8 (3)	C3—C4—C5—C8	179.1 (2)
N1—C1—C2—C3	−179.68 (19)	C5—C4—C3—C2	0.5 (4)
C6—C1—C2—C7	177.4 (2)	C1—C2—C3—C4	−0.1 (3)
N1—C1—C2—C7	−1.4 (3)	C7—C2—C3—C4	−178.5 (2)
C2—C1—C6—C5	1.3 (3)		
